# Motherhood in the Time of Coronavirus: The Impact of the Pandemic Emergency on Expectant and Postpartum Women’s Psychological Well-Being

**DOI:** 10.3389/fpsyg.2020.567155

**Published:** 2020-10-26

**Authors:** Sara Molgora, Monica Accordini

**Affiliations:** Department of Psychology, Catholic University of the Sacred Heart, Milan, Italy

**Keywords:** expectant mothers, new mothers, COVID-19, restrictive measures, psychological well-being

## Abstract

The birth of a child is a critical and potentially stressful experience for women, entailing several changes both at the individual and interpersonal level. This event can lead to different forms of distress, ranging in intensity and duration. Many studies highlighted medical, psychological, and social variables as risk factors potentially influencing the onset or aggravation of perinatal maternal conditions. The current pandemic emergency and the restrictive measures adopted by local governments to prevent the spread of the coronavirus infection may negatively affect mothers-to-be and new mothers potentially increasing the likelihood of anxiety, depressive or post-traumatic symptoms to develop. Moreover, the forced quarantine combined with the limited access to professional or family support may increase feelings of fatigue and isolation. The present study aims to investigate women’s psychological well-being during pregnancy and in the first months after childbirth, integrating the evaluation of some traditionally studied variables with the specificities of the current situation. 575 Italian women have been administered an online self-report questionnaire assessing the presence of anxiety disorders, depressive and post-traumatic symptoms as well as the expectations toward childbirth (for mothers-to-be) or the subjective experience of childbirth (for postpartum women). Findings revealed a higher percentage of women than that reported in the literature scored above the clinical cut-off both during pregnancy and postpartum on a series of measures of psychological well-being, thus demonstrating that this period was perceived as particularly challenging and stressful and had significant impact on the women’s well-being. Moreover, some socio-demographic, medical, and pandemic-related variables, especially the lack of presence and support from one’s partner during labor and delivery as well as in the first days postpartum was found to predict women’s mental health. These findings suggest the need for developing specific interventions targeted at women who cannot benefit from the support of their partners or family.

## Introduction

The birth of a child represents a critical experience in a woman’s life, entailing several changes both at the individual (physical and psychological) and interpersonal level (Lawrence et al., 2008; Guzzo and Hayford, 2020). This experience may negatively impact on individual well-being, leading to several forms of distress and/or diseases including anxiety, mood and post-traumatic stress disorders (Paulson and Bazemore, 2010; Meltzer-Brody et al., 2017; Mohamied, 2019; Pellowski et al., 2019).

Most of the literature has focused on postpartum adjustment, however a growing body of research has progressively investigated women’s well-being also during pregnancy (Molgora et al., 2020a). Both pregnancy and childbirth can be considered as potentially stressful, even traumatic, events requiring women to cope with changes in their lifestyles, habits, and even in their self-image and identity (Molgora et al., 2018). Moreover, it is important to underline that the psychological well-being of expecting mothers can influence their subjective experience of childbirth (Molgora et al., 2020b), as well as the medical-obstetric aspects of labor and delivery, for example in terms of prolonged labor and greater likelihood of undergoing operative deliveries or cesarean-sections (Fenaroli et al., 2016).

### Psychological Well-Being of Expectant Mothers

Previous research on pregnant women has extensively studied the impact and prevalence of antenatal anxiety and depression reporting high rates of such conditions across various countries (Biaggi et al., 2016; Falah-Hassani et al., 2017; Nasreen et al., 2018; van de Loo et al., 2018). In this respect, although differences have been reported across studies due to the methodological approaches used, the prevalence of anxiety disorders among pregnant women has been found to be approximately 15% (Dennis et al., 2017). Antenatal depression, on its side, has been reported to affect 10–20% of expectant mothers—depending on the different cut-off scores used in the studies (Zaers et al., 2008; Pampaka et al., 2018; Sunnqvist et al., 2018).

Furthermore, during pregnancy many women develop a severe fear of childbirth; this latter is a clinical condition characterized by several symptoms (sleep disorders, panic attacks, etc.) which greatly impair women’s ability to conduct their everyday life while also negatively affecting their ability to cope with labor and childbirth (Fenwick et al., 2009; O’Connell et al., 2017; Molgora et al., 2018). Although past research on this topic reported a great variability in prevalence rates, according to different cut-off scores and other methodological choices, the meta-analysis by O’Connell et al. (2017) estimated a prevalence of severe (i.e., clinical) fear of childbirth among 14% of pregnant women.

Several variables have been considered to be associated with fear of childbirth and overall maternal well-being, in this respect, different risk factors have been detected, both at an individual and interpersonal level. Some demographical dimensions (e.g., age, level of education, parity, etc.) have been investigated with contrasting results. For example, some studies reported multiparous mothers to be at higher risk of distress (Lainer and Jonson-Reid, 2014; Bassi et al., 2017), whereas other studies found nulliparous women to be at greater risk for severe fear of childbirth (Rouhe et al., 2008). Furthermore, distressing experiences occurring before or during pregnancy (e.g., serious physical illness, loss of a loved one, etc.) as well as a variety of medical-obstetric variables prior to and during pregnancy (e.g., previous miscarriages, high risk pregnancy, etc.) have been found to impact on pregnant women’s mental health (e.g., Devlin et al., 2016; Furtado et al., 2018). Again, a personal history of mental illness prior to pregnancy has been reported to increase the risk of developing a psychological disorder during pregnancy, thus underling the continuity of psychological disorders (Cox et al., 2014; Biaggi et al., 2016).

At an interpersonal level, many studies have highlighted the association between expectant mothers’ psychological well-being and relational variables, specifically quality of the couple relationship and social support, both during pregnancy and delivery (Lukasse et al., 2014; Figueiredo et al., 2018; Poggi et al., 2018): the lack of social support both from one’s partner or extended network has been found to be one of the most important predictor of antenatal anxiety and depression (Biaggi et al., 2016). Specifically, partner’s support resulted to mediate the relation between pregnant women’s concerns and their psychological well-being (Ilska and Przybyła-Basista, 2017). Moreover, the social support perceived by mothers during pregnancy represented a protective factor against postpartum depression while also reducing the negative aspects of the experience of childbirth (Tani and Castagna, 2017).

### Psychological Well-Being of Postpartum Women

Research on postpartum women has identified a wide range of clinical conditions that may impact on their well-being following childbirth: from the more common baby blues, that is a physiological and transitory condition (Rezaie-Keikhaie et al., 2020), to more severe conditions, such as anxiety and mood disorders, puerperal psychosis, and post-traumatic stress disorder (Paulson and Bazemore, 2010; Meltzer-Brody et al., 2017; Mohamied, 2019; Pellowski et al., 2019). Specifically, the prevalence of anxiety spectrum disorders has been found to be around 10% (15% for anxiety symptoms) (Dennis et al., 2017), whilst depressive symptoms have been reported in 10–22% of mothers, depending on the different cut-off scores used in the studies (Zaers et al., 2008; Pampaka et al., 2018; Sunnqvist et al., 2018). Moreover, a considerable number of women have been reported to have had a negative childbirth experience, specifically, the study by King et al. (2017) reported that about one-third of the women in the sample described their childbirth as very negative or traumatic. Finally, postpartum PTSD was found to be around 6% at 6 weeks postpartum and around 15% at 6 months postpartum (Zaers et al., 2008), while another study (Yildiz et al., 2017) reported a 4% mean prevalence of postpartum PTSD in community samples, and a 18.5% prevalence among high-risk groups.

Several variables have been analyzed in association with maternal psychological health postpartum. First, mothers’ subjective experience of childbirth has been found to predict their postpartum well-being (MacKinnon et al., 2017; Molgora et al., 2020b). The quality of this experience can be shaped by numerous variables (e.g., medical, demographical, psychological, social) that have been analyzed as risk/protective factors (Fenaroli et al., 2016, 2019). For example, fear of birth, the use of epidural analgesia, and the duration of the expulsive phase have been found to significantly predict the mothers’ experience of birth (Fenaroli et al., 2019). Considering the interpersonal dimension, women who received more intra-partum support and care, especially from their partner, had shorter labors, a more positive birthing experience characterized by higher levels of satisfaction, and a better postnatal psychological functioning (Collins et al., 1993; Hodnett, 2002; Hodnett et al., 2011; Michels et al., 2013). Mothers’ psychological health is a key variable that needs to be carefully considered given the impact of maternal well-being on the quality of their first interaction with the newborn, on the baby development as well as on the overall family functioning (Choi et al., 2017; van de Loo et al., 2018).

### Psychological Well-Being During the Covid-19 Pandemic

Starting from December 2019, the Covid-19 pandemic has affected several nations around the world. A growing number of studies have investigated the psychological impact of this pandemic on the general population reporting an increase of emotional distress and social disorders following the outbreak, especially among women (e.g., Rajkumar, 2020; Thapa et al., 2020). In particular, a previous Italian nationwide study, carried out during the first period after the Covid-19 outbreak and aimed at investigating the social and psychological impact of the pandemic, reported a widespread decrease in psychological well-being, especially among women younger than 50 and with previous health risk factors (Favieri et al., 2020).

Specifically, the outbreak and the restrictive measures enforced by local governments to prevent the spread of the coronavirus infection can be considered as additional major stressors for mothers-to-be and postpartum women, whose vulnerability is potentially increased by this specific situation, thus leading to detrimental effects on their psychological well-being (Thapa et al., 2020). Most of the studies published in the last months on the effect of the Covid-19 pandemic on pregnancy and childbirth have mainly focused on medical conditions and risk factors and have attempted to pinpoint the measures and clinical recommendations required to contain the Covid-19 spread (e.g., Chen et al., 2020; Liang and Acharya, 2020). The scant literature that has addressed the psychological impact of the Covid-19 pandemic on expectant and postpartum women has found higher levels of anxiety and depressive symptoms among participants when compared to similar cohorts assessed before the outbreak (Ceulemans et al., 2020; Lebel et al., 2020; Liu et al., 2020; Patabendige et al., 2020). An Italian study investigating the psychological effects of the pandemic on pregnant women found a significant change in their expectations toward pregnancy and childbirth as well as an increase in their concerns and distress, especially if they had a previous history of psychological distress in press (Ravaldi et al., 2020). Furthermore, women showed significant concerns mainly related to the risk of coronavirus infection and to the fear of consequent complications for themselves and the fetus and were more likely to complain about insufficient antenatal support (Haruna and Nishi, 2020). In this respect, it is important to underline that the pandemic has had an effect on both antenatal care as well as on birth management and postpartum care (Coxon et al., 2020). During the pandemic, and especially during the lockdown period, pregnant and postpartum women have experienced a limited access to their formal and informal support network, thus facing new and unforeseen struggles potentially putting their mental health in jeopardy. The presence of a support network has, in fact, been widely recognized as a crucial protective factor for prospective mothers and postpartum women alike (Friedman et al., 2020; Huschke et al., 2020). The importance of such support is particularly relevant in family centered cultures, such as the Italian one, where – also thanks to the geographic proximity – children tend to turn to their parents for support also during adulthood (Scabini, 2000). For example, an Italian study by Agostini et al. (2014) found that women with low levels of social support were more likely to experience depressive symptoms.

### The Italian Situation

Italy has been the first European–and more generally Western country–to report some coronavirus cases. The outbreak occurred in late February in Northern Italy and, since then, the local government has adopted a series of restrictive measures to contain the pandemic that have been considered among the strictest in Europe.

In particular, on February 23 the Italian government issued the first decree (D.L. n.6, 23/2/2020) establishing quarantine red-zones around the most severely hit Northern cities. People living in the area were forbidden to exit while those living outside were denied access, moreover, people were invited to remain at home unless absolutely necessary. On March 9, a new government order (D.P.C.M. 9/3/2020) was issued, extending the quarantine zone to the whole national territory: gatherings of more than two people were prohibited and companies invited to encourage working from home. On March 11 all shops and factories selling or manufacturing non-necessary goods were closed. On March 20 a new government order (D.P.C.M. 20/3/2020) enforced further limitations: parks and gardens were closed to the public and physical activity was allowed only within the premises of one’s house. On April 10 the Italian prime minister announced that the above-mentioned restrictions would be enforced until May 3, the official end of the first phase of the emergency.

Besides these restrictions that affected the whole population forcing millions of people to change their habits, routines and lifestyle, pregnant and postpartum women had to face further limitations. While such restrictions varied greatly across regions and even within the same region, all the hospitals adopted some sort of limitations that impacted on pregnant and postpartum women. More specifically, women could not attend antenatal classes; both non-urgent pre- and post-natal screenings and checkups were canceled or postponed; women had to attend visits unaccompanied; hospital access to fathers at postpartum was either completely forbidden or greatly limited and in many cases prospective fathers were denied access during childbirth and delivery; finally, some women were denied access to epidural analgesia due to medical staff being occupied with facing the pandemic emergency.

### The Current Study

The present study aims at investigating Italian women’s psychological well-being during pregnancy and in the first months after childbirth. To reach this aim, our research integrates the evaluation of some traditionally studied variables that are known to influence childbirth and delivery with the investigation of some variables strictly related to the coronavirus emergency. In particular, we will consider some psychological outcomes (i.e., anxiety symptoms, depressive symptoms and fear of childbirth for pregnant women, and anxiety symptoms, depressive symptoms, post-traumatic stress disorders and subjective experience of labor and delivery for postpartum women) and assess the role of several factors (anamnestic and personal information, relational variables, contextual variables), both related and unrelated to the current emergency, in predicting these outcomes.

In particular, it is possible to assume that the usual distress several women undergo due to the important and critical experience of motherhood would be amplified by the pandemic. Indeed, this situation has already been demonstrated to have had a negative impact on prospective mothers’ and postpartum women’s psychological well-being. Specifically, previous studies on Italian women found higher level of anxiety symptoms (Saccone et al., 2020) along with increased concerns and fears about childbirth (Ravaldi et al., 2020) among pregnant women. Moreover, international studies found greater rates of depression, anxiety and stress among mothers of infants and young babies (Cameron et al., 2020). Considering these preliminary results, we can hypothesize that pregnant and postpartum women are at greater risk of developing anxiety, depression, or post-traumatic symptoms. Such adverse outcomes may occur due to being infected or having a family member infected with the virus, experiencing the hospitalization or the death of a loved one, the increase of household and caregiving tasks, isolation, financial instability, domestic violence or abuse following the quarantine, and uncertainty about the future (Shah et al., 2020).

## Materials and Methods

### Participants and Procedure

The present cross-sectional study involved 575 Italian expectant mothers and postpartum women. Participants were recruited through social media (i.e., Facebook and Instagram) and invited to fill-in an online anonymous questionnaire. Data were collected between March 1, 2020 and May 3, 2020 (the so-called phase 1 of lockdown, characterized by the exceptionally strict measures adopted to prevent coronavirus spread). Inclusion criteria were: being above 18 years of age, being fluent in Italian, and being either pregnant or having had a baby for less than 6 months.

After receiving a short presentation of the research goals and proving their informed consent, all participants were asked to fill in a questionnaire on the platform Qualtrics. Informed consent forms and all the study materials have been approved by the Institutional Review Board of the authors’ institution.

Seven hundred eighteen questionnaires were collected: 130 were excluded because of missing or incomplete data, another 13 women were excluded because they did not fit inclusion criteria (i.e., their child was older than 6 months). The final sample comprised 575 women, 389 pregnant women and 186 postpartum women.

### Measures

In order to study the impact of the Covid-19 pandemic outbreak and of the above-mentioned restrictive measures on pregnancy and childbirth, researchers developed a questionnaire that was disseminated online. Besides gathering socio-demographic data (i.e., age, education, job title, parity, etc.), data on psychological well-being (i.e., number and type of stressful life events experienced at the time of data collection, such as economic problems, work problems, bereavements; number and type of previous psychological disorders (such as eating disorders, drug addition, alcoholism), and basic information regarding the pregnancy (i.e., mode of conception, number and type of complications occurred, type of pregnancy, etc.) and delivery (i.e., type of delivery, occurrence of health risk for the mother or the baby, access to epidural analgesia, etc.) were collected.

Furthermore, the questionnaire also included some questions specifically pertaining the Covid-19 emergency. Specifically, participants were asked if they or one of their family members had resulted positive to Covid-19, if their partners continued to commute to work during lockdown, if their partners were present (or if they believed they would be present in the case of pregnant women) at childbirth and if they could (or they believed they would be allowed to) visit them at the hospital. Moreover, mothers were asked about their access to epidural analgesia as well as to some form of family or professional support (specifically, the question asked: “When your baby will be born, will you receive help from someone?” and the possible answers were: my mother, my father, my mother-in-law, my father-in-law, another relative, a professional nurse, a doula, a babysitter, other). All the questions included in the questionnaire (except one, which has not been analyzed in the current study) were multiple choice. Lastly, the questionnaire included several measures (see below) of maternal well-being.

More specifically, all the women who took part in the research completed the following instruments:

*State-Trait Anxiety Inventory–STAI, Y form* (Spielberger et al., 1983; Italian validated version by Pedrabissi and Santinello, 1989).

This instrument is largely used to measure state and trait anxiety. It is composed of 40 items on a four-point Likert scale (20 items for trait anxiety and 20 items for state anxiety), with a total possible range of scores of 20–80 with higher scores indicating higher anxiety levels. Internal consistency was good for both the state (Cronbach’s alpha = 0.95 for both expectant and postpartum women) and trait (Cronbach’s alpha = 0.91 and 0.92, respectively for expectant and postpartum women) subscales. Based on previous studies on the Italian pregnant population (Giardinelli et al., 2012; Vismara et al., 2016), a cut-off score of 40 or higher was used to identify both state and trait clinical anxiety.

*Edinburgh Postnatal Depression Scale–EPDS* (Cox et al., 1987; Italian validated version by Benvenuti et al., 1999).

This instrument, originally developed to screen for postpartum depression has been validated and is currently used also during pregnancy (Kozinszky and Dudas, 2015). The scale is composed of 10 items on a four-point Likert scale, with a 0–30 total possible range of scores: the higher the score, the higher the depressive symptoms. Internal consistency was good (Cronbach’s alpha = 0.87 for both expectant and postpartum women). According to Gibson et al. (2009), a cut-off value of 13 or higher was used to distinguish clinical depression.

Pregnant women were administered the following instrument:

*Wijma Delivery Expectancy Questionnaire–WDEQ(A)* (Wijma et al., 1998; Italian validated version by Fenaroli and Saita, 2013).

This instrument measures expectations regarding childbirth, and in particular fear of childbirth, through 33 items on a six-point Likert scale. In the present study, we used the validated 14-item Italian version of the scale that has been proven to work better with the Italian population (Fenaroli and Saita, 2013; Molgora et al., 2018, 2020a). For this version, the total score ranges from 0 to 70: the higher the score, the more negative the expectations and the greater the fear toward childbirth. Internal consistency was good (Cronbach’s alpha = 0.90). Although to date there is no unique cut-off value identified as the gold standard to screen for clinical (i.e., severe) fear of childbirth, based on some studies that reported intense fear as falling in the top quartile of the continuous measure (Fenwick et al., 2009), values equal or above 35 were considered indicators of a severe fear (Molgora et al., 2020a).

Postpartum women were administered the following instruments:

*Wijma Delivery Experience Questionnaire–WDEQ(B)* (Wijma et al., 1998; Italian validated version by Fenaroli and Saita, 2013).

This instrument measures the childbirth experience through 33 items on a six-point Likert scale. As for the WDEQ(A), in the present study, we used the validated 14-item Italian version of the scale, whose total score ranges from 0 to 70: the higher the score, the more negative the subjective experience. Internal consistency was good (Cronbach’s alpha = 0.91). Similarly to the antenatal version of the instrument, a total score of 35 or higher was considered as the cut-off to distinguish severe fear.

*Perinatal PTSD Questionnaire–PPQ* (DeMier et al., 1996; Callahan et al., 2006; Italian not validated version by Di Blasio et al., 2009, 2015; Ionio and Di Blasio, 2014).

This instrument was developed to assess post-traumatic symptoms related to the experience of childbirth; specifically, the scale measures intrusiveness or re-experiencing, avoidance behaviors, hyperarousal or numbing, as well as feelings of guilt. The original version is composed of 14 items measured on a dichotomous scale while the revised version uses a five-point Likert scale, with a total score ranging from 0 to 56, with higher scores indicating more severe PTSD symptoms. This study uses the modified version of the scale which, although not validated in Italian, has already been used in a previous study (Di Blasio et al., 2015) showing good consistency (Cronbach’s alpha ranged from 0.85 to 0.88). Internal consistency was good (Cronbach’s alpha = 0.78). According to Callahan et al. (2006), a score of 19 or higher identifies high-risk mothers.

### Data Analyses

First, descriptive statistics were performed for each of the two samples separately: mean scores were calculated for age and frequencies were computed for variables such as education, job title, psychological disorders, stressful life events, etc. Measures of anxiety, depression, postpartum PTSD, and childbirth experience were dichotomized using cut-off values suggested by previous relevant studies. Those cut-off values were used to determine the percentage of women falling above the clinical range for each of the constructs under analysis.

Secondly, the expectant and postpartum mothers group were compared with respect to the socio-demographic and medical-obstetric variables as well as with respect to scores on depression and anxiety. Independent samples *t*-tests, Mann–Whitney and chi square tests were used according to the type of variable and its measurement scale.

After these preliminary steps, the two samples were analyzed separately given both the distinctive features characterizing the two conditions as well as the difference in sample size. Specifically, chi-square tests were run on both samples to investigate the relation between measures of anxiety, depression, postpartum PTSD, expectations toward or experience of childbirth and some demographic or medical-obstetric variables that are known to influence such relation. Further chi-square tests were performed on both the expectant and the postpartum mothers samples to assess the differences between women falling above the clinically significant range for the above-mentioned scales (i.e., STAI, WDEQ(A) and (B), PPQ, and EPDS) and those falling below the cut-off with respect to variables measuring the impact of the restrictions adopted to prevent the spread of coronavirus. Dummy coded versions of the dependent variables (with zero indicating the absence of a clinically relevant condition and one indicating a clinically relevant score) were used in the above-mentioned analyses. *p* values resulting from these analyses have been corrected for multiple testing using the Holm–Bonferroni method.

Finally, a set of hierarchical logistic regressions were performed on the expectant and postpartum women sample separately. Specifically, total scores for STAI, EPDS, PPQ, WDEQ(A) and (B) were entered as dependent variables in the various equation models performed. Unlike we did for chi-square tests, in this case total scores for anxiety, depression, fear and experience of childbirth, as well as postpartum PTSD were used. The choice of using continuous instead of dummy variables was determined by the willingness to retain the maximum data variability. Each of the above-mentioned variables was regressed against a set of independent variables including demographic, medical and obstetric data, as well as data regarding the impact of the pandemic emergency on the women’s life. Specifically, a three-step multiple regression procedure was used in which each criterion variable was regressed on the predictor variables in blocks. Demographic factors as well as data concerning the women’s medical and psychological general condition (i.e., number of stressors, number of previous psychological conditions) were entered in the first step, followed by dimensions related to pregnancy (e.g., number of complications, etc.) and childbirth (e.g., presence of risks for the mother or the child’s health, etc.). Finally, factors measuring the impact of the Covid-19 pandemic on the women’s experience or expectations (e.g., perceived likelihood of father being present at delivery and during hospital stay or actual presence of father at delivery and hospitalization) were entered in the third block. Our choice was motivated by the fact that the first block contained basic variables regarding the women’s demographics and well-being irrespectively of their condition, the second block included all the variables related to pregnancy and childbirth while the third block incorporated all the measures of the effect of Covid-19 pandemic on expectant and postpartum women.

## Results

### Descriptives and Comparison Between Expectant Mothers and Postpartum Women

Pregnant women’s mean age was 32.9 (SD = 4.3), 63.2% (246) of women were married to the father of their child, 35.7% (139) were cohabiting and the remaining 0.8% (3) were either single or separated/divorced. 53.2% (207) of the pregnant women in our sample did not have other children whereas 45.2% (176) were multiparous. Only a very small proportion of women (4.6%, 18) claimed that they or one of their immediate family members had resulted positive to coronavirus. Most of the women’s partners (62.1%) were working from home while the remaining 38.8% were continuing to commute to work during lockdown. With respect to medical appointments and checkups, 80.5% of the women in our sample declared that they were undergoing regular doctor visits while 15.2% stated that their appointments were canceled or rescheduled due to the Covid-19 emergency. Similarly, 8.7% (34) of the women in our sample were told that they could not use epidural analgesia due to the lack of medical staff caused by the coronavirus outbreak. Finally, 7.5% of the women were told their partners could not be present during childbirth and 47.0% were unsure about their partners’ presence at delivery due to the restrictive measures adopted to contain the Covid-19 spread. Similarly, 13.9% of women believed their partners would not be allowed to visit them during hospitalization.

With regards to postpartum women, mean age was 33.01 (SD = 4.19), 109 (58.6%) women were married to the father of their child while the remaining 77 (41.4%) were cohabiting, 53.8% (100) of the women did not have other children whereas 45.2% (84) had at least one more child. Similarly to what happened for pregnant women, only six (3.2%) postpartum women stated that they or one of their family members had resulted positive to Covid-19. With regards to fathers, 34.9% of women declared that their partners continued working during lockdown, 21% declared that their partners were not admitted during childbirth and 10.8% stated that they were not admitted during hospital stay.

Other data regarding women’s education, job title, number and type of current stressors (independent from the coronavirus emergency), number and type of previous mental disorders, and number and type of complications during pregnancy are reported in Table 1.

**TABLE 1:** Descriptive statistics for pregnant women and postpartum women.

	Pregnant w.		Postpartum w.	
	*N*	%	*N*	%
**Education**				
Middle school diploma	19	4.9	6	3.2
Professional course license	10	2.6	3	1.6
High school diploma	125	32.1	53	28.5
University degree	173	44.5	96	51.6
Postgraduate Masters/PhD	52	13.4	25	13.4
Other	9	2.3	3	1.6
**Job title**				
Freelance professional	61	15.7	26	14
Employed	234	60.2	120	64.5
Unemployed	39	10.0	12	6.5
Housewife	28	7.2	13	7
Student	4	1.0	3	1.6
Other	22	5.7	11	5.9
**Stressors (not related to Covid-19)**				
Financial problems	62	15.9	22	11.8
Work problems	70	18.0	24	12.9
Personal health problems	2	0.5	2	1.1
Health problems of a family member	25	6.4	19	10.2
Death of a family member/close friend	18	4.6	9	4.8
Other	25	6.4	10	5.4
**Number of stressors**				
1	86	22.1	41	22
2	40	10.3	10	5.4
3	7	1.8	2	1.1
**Previous psychological disorders**				
Mood disorders	81	20.8	43	23.1
Anxiety disorders	158	40.6	71	38.2
Eating disorders	59	15.2	32	17.2
Alcohol abuse	7	1.8	7	3.8
Drug abuse	5	1.3	8	4.3
Other	3	0.8	3	1.6
**Number of previous psychological disorders**				
1	120	30.8	68	43.0
2	64	16.5	27	36.6
3	14	3.6	5	14.5
4	2	0.5	3	2.7
5	3	0.8	3	1.6
**Complications during pregnancy**				
Risk of miscarriage	46	11.8	28	15.1
Ectopic pregnancy	6	1.5	7	3.8
Placental abruption	29	7.5	13	7,0
Hyperemesis gravidarum	94	24.2	38	20.4
Gestational diabetes	27	6.9	21	11.3
Other	23	5.9	17	9.1
**Number of complications**				
1	125	32.1	62	33.3
2	32	8.2	17	9.1
3	7	1.8	2	1.1
4	1	0.3	1	0.5

The two samples (i.e., pregnant women and postpartum women) did not differ with regards to the socio-demographic variables investigated. Specifically, no difference between the two groups was found for age (M_*pregnant women*_ = 32.90, SD = 4.32; M_*postpartum*_ = 33.01, SD = 4.19) [*t*(569) = −0.291, *p* = 0.37], education [*U* = 34126.00, *p* = 0.26], job title [χ^2^(5, 573) = 2.82, *p* = 0.73], and whether they had other children [χ^2^(1, 567) = 0.01, *p* = 0.95].

Moreover, the two groups did not differ with regards to number of psychological disorders [*U* = 34857.00, *p* = 0.45] or stressful life events [*U* = 32082.00, *p* = 0.14] experienced in their lives and to whether they, or one of their immediate family members, had tested positive to Covid-19 [χ^2^(1, 561) = 0.524, *p* = 0.47].

With reference to anxiety, 64.0% (249) of expectant mothers and 57.7% (98) of postpartum women scored above the clinically significant range for state anxiety, data regarding trait anxiety revealed that 44.0% (171) of pregnant women and 46.2% (86) of postpartum fell above the cut-off score. Moreover 34.2% (133) of expectant women and 26.3% (49) of postpartum women had clinically significant levels of depression as measured by the EPDS scale. 31.7% of postpartum women reported a negative childbirth experience while 51.2% of expectant women had negative expectations regarding birth. Finally, postpartum PTSD scores were above the cut-off value in 16.7% of cases. Independent samples *t*-tests comparing state and trait anxiety and depression scores in pregnant women vs. postpartum women did not reveal any significant difference between the groups.

Although no differences between expectant mothers and postpartum women were found for anxiety and depressive symptoms, subsequent analyses have been run separately for each of the two samples given the specificity of each condition and the difference in sample size.

### Psychological State of Expectant Mothers: Non-Covid Related Risk Factors

With regards to pregnant women, a set of chi square analyses were performed to explore the relationship between state anxiety, trait anxiety, fear of childbirth, and depression and a number of variables that are known to influence such relation (i.e., the presence of previous child(ren); the presence and number of past psychological disorders, the number of stressors experienced at the time of data collection, the number of complications occurred during pregnancy, etc.).

Pregnant women having more than one child [χ^2^(1, 342) = 10.35, *p* = 0.008] and those having suffered from previous psychological disorders [χ^2^(5, 348) = 20.17, *p* = 0.008] were more likely to experience state anxiety. Similar results were found for EPDS scores, with multiparous women [χ^2^(1, 356) = 7.46, *p* = 0.05] and those having suffered from previous disorders [χ^2^(5, 362) = 21.3, *p* = 0.009] being more likely to be psychologically depressed. With regards to trait anxiety, only the presence of previous psychological disorders scored significantly, with pregnant women suffering from two or three previous disorders being more likely to fall above the clinical cut-off [χ^2^(5, 345) = 45.48, *p* < 0.000]. With regards to fear of childbirth, none of the above-mentioned variables was significant.

### Psychological State of Expectant Mothers: Covid-Related Risk Factors

Following, further chi square tests were run to investigate the relation between the above said measures of psychological disorders (i.e., STAI state, STAI trait, WDEQ(A), and EPDS) and some variables connected to the Covid-19 pandemic and the subsequent restrictions imposed on the population. Specifically, relations between depression, childbirth expectations, state and trait anxiety and perceptions regarding access to epidural analgesia, postpartum support, possibility for fathers to be present at delivery and to visit the mother during hospitalization were investigated, together with father going vs. not going to work regularly during lockdown. Results showed that pregnant women who believed their partner could not be present at childbirth along with those who were unsure about the father being allowed to enter the delivery room were more likely to suffer from state anxiety [χ^2^(2, 348) = 15.44, *p* < 0.000] and to have intense fear of childbirth [χ^2^(2, 364) = 9.08, *p* = 0.007]. Similarly, women who believed their partner would be denied visitation and those who were not sure whether their partners would be allowed access to the hospital rooms were more likely to fall in the clinically significant range for state anxiety [χ^2^(2, 337) = 12.99, *p* = 0.012]. Lastly, pregnant women who believed they would not have access to any form of family or professional support after childbirth were more likely to suffer from state anxiety [χ^2^(1, 347) = 8.01, *p* = 0.025].

### Psychological State of Postpartum Women: Non-Covid Related Risk Factors

Similar analyses were conducted also on the postpartum women sample. More specifically, dummy scores for depression, quality of childbirth experience, postpartum PTSD, state and trait anxiety were measured against number of stressful events experienced in ones’ life, number of previous psychological disorders, presence of children, number of complications during pregnancy, access to epidural analgesia, presence of complications at childbirth for the mother or the child. Results showed that new-mothers who had experienced psychological disorders earlier in their lives were more likely to suffer from trait anxiety [χ^2^(5,166) = 38.25 *p* = 0.000], postpartum depression [χ^2^(5, 174) = 23.00, *p* = 0.000] and postpartum PTSD [χ^2^(5, 154) = 12.11, *p* = 0.007]. Moreover, mothers who experienced two or three complications during pregnancy were more likely to fall in the clinically significant range for postpartum PTSD [χ^2^(4, 154) = 9.36, *p* = 0.05] and to have perceived childbirth as a negative event [χ^2^(4, 179) = 13.58, *p* = 0.009]. The same was true for mothers who experienced some sort of health risk during delivery: these women were more likely to develop a postpartum PTSD [χ^2^(1, 154) = 4.95, *p* = 0.026] and to have negative memories of childbirth [χ^2^(1, 179) = 8.57, *p* = 0.033].

### Psychological State of Postpartum Women: Covid-Related Risk Factors

As it was the case for pregnant women, relations between depression, childbirth experience, postpartum PTSD, state and trait anxiety and Covid-19-related variables were also explored. In this case, women whose partners had not been present during delivery were more likely to experience both clinically significant state [χ^2^(1, 167) = 4.45, *p* = 0.035] and trait anxiety [χ^2^(1, 166) = 6.84, *p* = 0.009] as well as to develop a postpartum PTSD [χ^2^(1, 154) = 4.58, *p* = 0.032]. Postpartum women whose partners continued working regularly during lockdown also showed a greater likelihood of suffering from state anxiety [χ^2^(1, 167) = 5.28, *p* = 0.022].

### Effects of Specific (Covid-Related) and Non-specific Factors on Pregnant Women

After having explored the relations between anxiety, depression, expectations/experience of childbirth, postpartum PTSD and a series of psycho-social and health related variables both independent from and connected to the current pandemic situation and the restrictions adopted by the Italian government to contain the coronavirus outbreak, we decided to run a set of separate hierarchical logistic regressions for each of the two samples to assess whether and to what extent such variables contributed to pregnant and postpartum women falling in the clinical vs. non-clinical range.

With regards to pregnant women, four separate hierarchical logistic regression were run to evaluate the prediction of state and trait anxiety, depression, and fear of childbirth from presence of other children, number of stressors in one’s life, number of previous disorders, complications during pregnancy, prospective access to epidural analgesia, fathers’ employment status (i.e., whether the prospective father was going to work regularly during lockdown), perceived likelihood of fathers being present at delivery and during hospitalization. In all the four regressions number of stressors, number of previous disorders and presence of other children were entered in the first block, number of complications and prospective access to epidural analgesia were entered in the second block, while Covid-19 related variables (i.e., father going to work regularly during lockdown, perceived likelihood of father being present at delivery and during hospitalization) were entered in the third block. Results are presented in Table 2.

**TABLE 2:** Pregnant women–Hierarchical Regression Analyses for STAI State, STAI Trait, EPDS, and WDEQ(A).

	Model	STAI State	STAI Trait	EPDS	WDEQ(A)
Block 1	Other children (0 = No, 1 = Yes)	0.005*	0.003*	0.046**	0.048**
	Stressor 1	0.118	0.121	0.279	0.824
	Stressor 2	0.030**	0.081	0.465	0.402
	Stressor 3	0.315	0.925	0.251	0.187
	Disorders 1	0.023**	0.000*	0.005**	0.059
	Disorders 2	0.000*	0.000*	0.000*	0.039**
	Disorders 3	0.000*	0.000*	0.007*	0.058
	Disorders 4	0.857	0.530	0.462	0.328
	Disorders 5	0.221	0.206	0.993	0.503
	R^2^	0.143	0.187	0.100	0.049
	F	6.179	8.43	4.28	2.00
Block 2	Other children (0 = No, 1 = Yes)	0.005*	0.003*	0.046**	0.043**
	Stressor 1	0.090	0.114	0.350	0.865
	Stressor 2	0.029**	0.103	0.558	0.453
	Stressor 3	0.325	0.986	0.284	0.193
	Disorders 1	0.018**	0.000*	0.005*	0.077
	Disorders 2	0.000*	0.000*	0.000*	0.049
	Disorders 3	0.002*	0.000*	0.009*	0.087
	Disorders 4	0.879	0.484	0.435	0.327
	Disorders 5	0.220	0.190	0.968	0.458
	Epidural analgesia (0 = No, 1 = Yes)	0.885	0.187	0.325	0.482
	Complications pregnancy 1	0.951	0.790	0.871	0.378
	Complications pregnancy 2	0.294	0.856	0.931	0.779
	Complications pregnancy 3	0.242	0.503	0.056	0.683
	R^2^	0.150	0.193	0.126	0.059
	ΔR^2^	0.007	0.006	0.026	0.010
	ΔF	0.672	0.598	2.034	0.713
Block 3	Other children (0 = No, 1 = Yes)	0.007*	0.004*	0.035**	0.198
	Stressor 1	0.141	0.117	0.049**	0.041**
	Stressor 2	0.023**	0.093	0.373	0.837
	Stressor 3	0.528	0.873	0.492	0.394
	Disorders 1	0.013**	0.000*	0.468	0.311
	Disorders 2	0.000*	0.000*	0.003*	0.058
	Disorders 3	0.002*	0.000*	0.000*	0.047**
	Disorders 4	0.859	0.457	0.011**	0.117
	Disorders 5	0.278	0.223	0.403	0.369
	Epidural analgesia (0 = No, 1 = Yes)	0.898	0.180	0.980	0.479
	Complications pregnancy 1	0.804	0.689	0.311	0.498
	Complications pregnancy 2	0.303	0.899	0.614	0.587
	Complications pregnancy 3	0.210	0.499	0.899	0.835
	Father at work (0 = No, 1 = Yes)	0.345	0.725	0.059	0.711
	Father at delivery (0 = No, 1 = Yes)	0.123	0.107	0.054	0.263
	Father during hospitalization (0 = No, 1 = Yes)	0.201	0.907	0.706	0.829
	R^2^	0.169	0.202	0.153	0.088
	ΔR^2^	0.019	0.008	0.027	0.029
	ΔF	2.433	1.116	3.57	3.65

*Significant at *p* < 0.01; **significant at *p* < 0.05.

With regards to state anxiety, all the three models tested proved to be statistically significant (*p* < 0.000), however, only presence of other children, presence of two stressful events in one’s life and presence of up to three previous disorders are capable of predicting anxiety scores across all the three models. Specifically, having more than one child, experiencing two stressful live events at the time of data collection and having suffered from up to three previous psychological conditions have all proven to be significantly and positively related to an increase in state anxiety (see Table 2). The final model including the above-mentioned predictors accounts for 17% of the variance, *F*(16, 341) = 4.13, *p* < 0.000.

Similar results were obtained when trait anxiety was considered, with all the three regression models being statistically significant and the third accounting for 20.2% of the total variance. Again, having more than one child and having suffered from up to three disorders in the past resulted in an increase in trait anxiety scores for pregnant women.

With regards to depression, those women having more than one child and having suffered from up to three psychological conditions had also higher depression scores in step one. Similar results were found for step two and three. Moreover, in step three women who did not believe their partner would be present during childbirth obtained significantly higher depression scores (β =−0.137, *p* = 0.013). The final model accounted for 15.3% of the variance, *F*(17, 355) = 3.59, *p* < 0.000.

Finally, when fear of childbirth was considered only the first (*p* = 0.038) and third (*p* = 0.014) models proved to be statistically significant. Presence of other children and of two previous psychological conditions were positively correlated with fear of childbirth both in step one and three of the equation; moreover, when “beliefs about the presence of the prospective father at childbirth” was added to the equation in the third block, it also proved to have a significant effect on WDEQ(A) scores. Specifically, women who believed their partner would be able to assist them during childbirth reported significantly lower level of fear (β = −0.154, *p* = 0.007). The final model (*p* = 0.014) accounted for 8.8% of the total variance.

### Effects of Specific (Covid-Related) and Non-specific Factors on Postpartum Women

Similarly to what had happened for pregnant women, four separate hierarchical regression were performed on the postpartum women’s sample. Total scores on state and trait anxiety, depression, experience of childbirth and postpartum PTSD were regressed on a linear combination of variables investigating socio-demographic data, pregnancy- and delivery-related issues. More specifically, presence of other children, number of stressors in one’s life at data collection, and number of previous psychological problems were entered in the first block; complications during pregnancy, access to epidural analgesia, presence of health risks for the mother or the child during delivery were added in the second block, and fathers’ employment status (i.e., whether new-fathers had gone to work regularly during lockdown), presence of father at delivery and hospitalization, presence of a support network after childbirth were analyzed in the third block. Results are shown in Table 3.

**TABLE 3:** Postpartum women–Hierarchical Regression Analyses for STAI State, STAI Trait, EPDS, WDEQ(B), and PPQ.

	Model	STAI State	STAI Trait	EPDS	WDEQ(B)	PPQ
Block 1	Other children (0 = No, 1 = Yes)	0.079	0.457	0.972	0.077	0.136
	Stressor 1	0.301	0.044**	0.598	0.267	0.025**
	Stressor 2	0.557	0.994	0.489	0.012**	0.647
	Stressor 3	0.253	0.252	0.224	0.239	0.793
	Disorders 1	0.006*	0.000*	0.002*	0.045*	0.066
	Disorders 2	0.000*	0.000*	0.000*	0.024*	0.000*
	Disorders 3	0.103	0.001*	0.057	0.981	0.247
	Disorders 4			0.486	0.638	
	R^2^	0.166	0.288	0.202	0.125	0.174
	F	4.366	8.82	5.02	2.91	4.29
Block 2	Other children (0 = No, 1 = Yes)	0.075	0.287	0.834	0.039	0.200
	Stressor 1	0.198	0.044**	0.474	0.385	0.053
	Stressor 2	0.411	0.772	0.524	0.022**	0.401
	Stressor 3	0.432	0.331	0.320	0.107	0.980
	Disorders 1	0.004*	0.000*	0.003*	0.088	0.062
	Disorders 2	0.000*	0.000*	0.000*	0.137	0.000*
	Disorders 3	0.088	0.002*	0.077	0.747	0.805
	Disorders 4			0.921	0.873	
	Complications pregnancy 1	0.031*	0.179	0.144	0.953	0.872
	Complications pregnancy 2	0.443	0.651	0.482	0.114	0.549
	Complications pregnancy 3	0.155	0.285	0.422	0.227	0.009*
	Complications pregnancy 4	0.183	0.224	0.986	0.812	0.819
	Epidural analgesia (0 = No, 1 = Yes)	0.402	0.264	0.528	0.067	0.687
	Health problems childbirth (0 = No, 1 = Yes)	0.345	0.922	0.144	0.322	0.013**
	Baby health problems childbirth (0 = No, 1 = Yes)	0.144	0.635	0.181	0.002	0.310
	R^2^	0.234	0.323	0.241	0.217	0.250
	ΔR^2^	0.069	0.035	0.039	0.092	0.077
	ΔF	1.884	1.078	1.13	2.609	1.98
Block 3	Other children (0 = No, 1 = Yes)	0.115	0.381	0.918	0.022	0.188
	Stressor 1	0.232	0.058	0.589	0.450	0.050**
	Stressor 2	0.407	0.783	0.593	0.015	0.478
	Stressor 3	0.470	0.348	0.284	0.083	0.811
	Disorders 1	0.007*	0.001*	0.004*	0.099	0.122
	Disorders 2	0.000*	0.000*	0.000*	0.225	0.001*
	Disorders 3	0.075	0.002*	0.080*	0.663	0.552
	Disorders 4			0.953	0.994	
	Complications pregnancy 1	0.051	0.219	0.152	0.955	0.896
	Complications pregnancy 2	0.521	0.755	0.526	0.100	0.413
	Complications pregnancy 3	0.173	0.299	0.483	0.176	0.011**
	Complications pregnancy 4	0.236	0.272	0.781	0.969	0.894
	Epidural analgesia (0 = No, 1 = Yes)	0.559	0.393	0.719	0.048	0.954
	Health problems childbirth (0 = No, 1 = Yes)	0.324	0.855	0.102	0.256	0.015**
	Baby health problems childbirth (0 = No, 1 = Yes)	0.229	0.519	0.299	0.001	0.188
	Father at work (0 = No, 1 = Yes)	0.411	0.790	0.308	0.154	0.261
	Father at delivery (0 = No, 1 = Yes)	0.212	0.215	0.049**	0.021**	0.018**
	Father during hospitalization (0 = No, 1 = Yes)	0.700	0.677	0.847	0.386	0.198
	Postpartum support (0 = No, 1 = Yes)	0.019**	0.732	0.255	0.220	0.619
	R^2^	0.248	0.332	0.275	0.250	0.284
	ΔR^2^	0.014	0.009	0.034	0.033	0.034
	ΔF	1.666	0.503	1.712	1.66	1.56

*Significant at *p* < 0.01; **Significant at *p* < 0.05.

With regards to state anxiety, all the three models proved to be significant (*p* < 0.000). Having suffered from up to two psychological conditions in the past significantly correlated with anxiety scores across the three models. Incremental of F scores revealed that the addition of postpartum support in step three resulted in a significant increase in *R*^2^ (Δ*R*^2^ = 0.01, Δ*F* = 1.67, *p* = 0.001). In other words, new-mothers who had suffered from previous psychological disorders in the past tended to be more anxious (β_*disorder1*_ = 0.221, *p* = 0.007; β_*disorder2*_ = 0.340, *p* = 0.000) whereas women who could count on the support of either a family member or a professional figure after delivery were significantly less anxious (β = −4.23, *p* = 0.019). Similar results were obtained for state anxiety: women having experienced up to three disorders in the past also had higher trait anxiety scores (β_*disorder1*_ = 0.265, *p* = 0.001; β_*disorder2*_ = 0.464, *p* = 0.000, β_*disorder3*_ = 250, *p* = 0.002). Moreover, in this case, the fact of having experienced one stressful life event at the time of data collection significantly impacted on anxiety levels only in model one (β = 0.140, *p* = 0.044). and two (β = 0.141, *p* = 0.044) but was not significant in model three (β = 0.136, *p* = 0.058) (see Table 3).

When depression was considered, analyses revealed that new-mothers who had experienced up to two previous conditions had higher EPDS scores and this was true in all the three models (third model β_*disorder1*_ = 0.229, *p* = 0.004; β_*disorder2*_ = 0.393, *p* = 0.000). At the same time, mothers who could count on the presence of their partner during delivery showed significantly lower levels of postnatal depression (β = −0.147, *p* = 0.049). The final model including the above-mentioned variables explained 27.5% of the variance.

With regards to childbirth experience, the presence of two concurrent stressful life events significantly impacts on WDEQ(B) scores and this variable plays a significant role across the three models (third model β_*stressor2*_ = 0.192, *p* = 0.015). On the contrary, the presence of up to two previous disorders resulted to be related to WDEQ(B) scores only in model one (β_*disorder1*_ = 0.158, *p* = 0.045; β_*disorder2*_ = 0.186, *p* = 0.024) while not being significant in model two (β_*disorder1*_ = 0.133, *p* = 0.088; β_*disorder2*_ = 0.123, *p* = 0.137) and three (β_*disorder1*_ = 0.129, *p* = 0.099; β_*disorder2*_ = 0.101, *p* = 0.225). While not being significant in the first model (β = 0.132, *p* = 0.077), the presence of other children becomes significant in the second (β = −0.132, *p* = 0.039) and third (β = −0.180, *p* = 0.022) model, showing that multiparous women have lower scores with respect to childbirth experience and, thus, perceive it as less negative. The same happens for epidural analgesia: while not being significant in the second step (β = −0.148, *p* = 0.067) of the model, this variable is significant in the third (β = −0.161, *p* = 0.048) (see Table 3). Therefore, the possibility of using epidural analgesia resulted in a significantly more positive evaluation of the experience of childbirth. The presence of problems for the baby’s health during delivery (β = 0.251, *p* = 0.001) was also positively correlated with an increased perception of childbirth as a negative event. Lastly, women who could count on the presence of their partner during delivery scored significantly lower than those who were alone (β = −3.068, *p* = 0.021). The final model accounted for 25% of the variance.

With regards to postpartum PTSD, the presence of one stressful life event at the time of data collection resulted significant only in step one (β = −0.175, *p* = 0.025) and three (β = −0.151, *p* = 0.050). The presence of two previous psychological conditions, on the contrary, was positively and significantly correlated to PPQ scores across the three models (first model β_*disorder2*_ = 0.386, *p* = 0.000; second model β_*disorder2*_ = 0.327, *p* = 0.000; third model β_*disorder2*_ = 0.312, *p* = 0.001). Moreover, the presence of three complications as well as the presence of problems for the baby’s health during delivery were also related to an increase in PPQ scores in model two (β_*complications3*_ = 0.227, *p* = 0.009); (β_*babyhealthproblems*_ = 0.207, *p* = 0.013) and three (β_*babyhealthproblems*_ = 0.204, *p* = 0.015). In other words, new-mothers who had experienced past psychological disorders, those who experienced numerous complications or whose baby’s health was at risk during childbirth were more likely to have higher postpartum PTSD scores. Finally, those mothers whose partners were present at childbirth scored significantly lower for postpartum PTSD (β = −0.230, *p* = 0.018). The final model including all the above-mentioned variables accounted for 28.4% of the variance.

## Discussion

Findings of this study confirmed that expectant and postpartum women’s psychological well-being can be influenced by several factors. Specifically, alongside some widely investigated dimensions that have proven to have an impact on mothers’ mental health (e.g., socio-demographic variables, previous psychological disorders, etc.), also specific pandemic-related factors (i.e., the restrictive measures enacted by the government to prevent and contain the spread of the coronavirus infection) have been found to shape the experience of motherhood, thus confirming our hypothesis as well as previously published studies findings (Ravaldi et al., 2020; Saccone et al., 2020; Thapa et al., 2020) that the responses enacted to prevent the spread of the virus are putting pregnant and postpartum women’s mental health in jeopardy.

First of all, a high percentage of women, both during pregnancy and in the postpartum, reported scores above the clinical cut-off for several measures of well-being. Moreover, such scores are higher than those reported in previous studies on the same topic. Although a certain variability among the various studies due to methodological choices has to be acknowledged, the prevalence of anxiety spectrum disorders was found to be around 15% in pregnant women and 10% in new-mothers (15% for anxiety symptoms) (Dennis et al., 2017); when our sample is considered, 44–64% (considering respectively trait and state anxiety) of pregnant women and 46.2% (trait anxiety)–57.7%(state anxiety) of postpartum women scored above the clinical cut-off for anxiety symptoms. Similar findings were found for depressive symptoms: depression rates reported in the literature range from 10 to 20% for pregnant women (depending on the different cut-off scores used in the studies) and from 10 to 22% for new-mothers (Zaers et al., 2008; Pampaka et al., 2018; Sunnqvist et al., 2018), however our participants showed clinically significant depressive symptoms in a much greater proportion: 34.2% during pregnancy and 26.3% at postpartum. Such figures are extremely relevant, especially if we consider that the highest possible threshold reported in the literature (i.e., 13 or above) has been used as the cut-off value for depression in the current study. When anxiety and depressive symptoms are compared, this study confirms that anxiety is usually more prevalent than depression among expectant mothers as already found in previous studies (Nasreen et al., 2018; van de Loo et al., 2018). However, while depression and anxiety symptoms are quite common among pregnant and postpartum women, the women in our sample were significantly more at risk for developing anxiety and depressive symptoms; these findings suggest that the pandemic and the measures adopted to fight its spread have had a negative impact on expectant and postpartum women’ well-being, thus constituting an additional risk factor for this specific population. We can hypothesize that social isolation, lack of support and control over one’s health may have negatively impacted women’s outcomes. Specifically, support both during pregnancy and in the postpartum have been found to be protective factors against depression (Collins et al., 1993; Hodnett, 2002; Hodnett et al., 2011; Michels et al., 2013; Goodman and Leiferman, 2016); similarly, an external health locus of control has been found to be associated with depressive symptoms in postpartum women (Richardson et al., 2012; Mollard, 2015).

Again, in our sample, fear of childbirth was above the cut-off value for more than half of expectant mothers, while 32% of postpartum women reported a negative childbirth experience. Research on this topic reported a great variety among fear of childbirth scores depending on the cut-off scores used and on some other methodological variables; however, the meta-analysis by O’Connell et al. (2017) estimated the presence of a severe (i.e., clinically significant) fear of childbirth in 14% of pregnant women. With regards to new-mothers, King et al. (2017) reported that about one-third of women describe their childbirth as very negative or traumatic, with a percentage that is in line with our study.

Finally, while postpartum PTSD scores ranged from 6% at 6 weeks postpartum to around 15% at 6 months postpartum in Zaers et al. (2008) study and from 4% for community samples to 18.5% in high risk samples as reported by Yildiz et al. (2017), our data show a percentage as high as 16.7%, thus assimilating the women in our sample to a high risk population. These findings suggest that fear, hyperarousal and other stress-related symptoms have been a constant in many women’s lives, potentially affecting their ability to take care of themselves and their child(ren).

Overall, women who gave birth or were pregnant during the acute phase of the current pandemic are at greater risk of developing depressive, anxiety or post-traumatic symptoms and of experiencing intense fear toward childbirth and this may lead to more complicated labors, greater pain at childbirth as well as to an impaired capacity of taking care of the baby while also affecting the overall family stability (Goodman and Leiferman, 2016, Molgora et al., 2020). In this scenario, both personal (especially the parity condition and the presence of previous disorders) and situational variables (especially the presence of the fathers both during labor and delivery, as well as during hospital stay) were found to predict mothers’ psychological mental health both during pregnancy and in the postpartum, distinguishing between clinical and non-clinical conditions on the investigated dimensions of psychological well-being.

Specifically, as for pregnant women, our results showed how multiparous women and those having suffered from previous psychological disorders were more likely to report anxiety and depressive symptoms, confirming previous studies (Cox et al., 2014; Lainer and Jonson-Reid, 2014; Biaggi et al., 2016; Bassi et al., 2017). Furthermore, expectant mothers who believed their partner could not be present at childbirth as well as those who believed their partner would be denied visitation were more likely to report higher levels of state anxiety symptoms or a severe fear of childbirth. Similarly, pregnant women who believed they would not have access to any form of family or professional support after childbirth were more likely to suffer from state anxiety. These results further confirm the crucial role social variables-especially the partner’s presence and support both during labor and delivery and in the postpartum (also in terms of mothers’ expectations)-have on expectant women’s psychological well-being (Lukasse et al., 2014; Biaggi et al., 2016; Figueiredo et al., 2018; Poggi et al., 2018).

As for postpartum women, our findings confirmed previous studies reporting that women who had experienced psychological disorders earlier in their lives were more likely to suffer from postpartum psychological distress and, in particular, anxiety, depression and PTSD (Cox et al., 2014; Biaggi et al., 2016). Moreover, postpartum women who experienced several complications during pregnancy were more likely to report postpartum PTSD and to report a negative experience of labor and delivery. This result is in line with other studies that have found an association between medical-obstetric dimensions of pregnancy and the subjective experience of childbirth as well as postpartum mental health (Devlin et al., 2016; Fenaroli et al., 2016, 2019; Furtado et al., 2018). Finally, women whose partners had not been present during delivery were more likely to experience both postpartum anxiety symptoms (state and trait) and PTSD thus underling the importance of (intra-partum and postpartum) partners’ support in preventing postpartum psychological distress (Collins et al., 1993; Hodnett, 2002; Hodnett et al., 2011; Michels et al., 2013; Tani and Castagna, 2017). On a similar note, previous studies (Kainz et al., 2010) have underlined the importance of prospective fathers or other caregivers during labor, explaining that these figures have a key role in infusing mothers with feelings of empowerment and well-being. It is within such framework that the association between fathers’ commuting to work and mothers’ anxiety can be understood: those women whose partners continued working regularly during lockdown showed a greater likelihood of suffering from state anxiety as they could not count on their daily support and they were constantly exposed to the risk of being infected thus adding an element of uncertainty and stress to an, already stressful, situation. In this respect, previous studies have demonstrated that women were extremely worried about themselves, their babies or one of their loved ones being infected with the virus and this resulted in an increased stress and fear (Cameron et al., 2020; Saccone et al., 2020).

Overall, these results underlined differences between primiparous and multiparous women, both during pregnancy and in the postpartum: women who already have other children seem to be more vulnerable than those who do not. We can suppose that the presence of other children made it more difficult for women to cope and manage everyday chores around the house, especially during the lockdown with all the schools being closed and the didactic activities provided at a distance. Due to the extremely severe measures adopted by the government, Italian mothers often found themselves to simultaneously take care of the house, help their children with school and carry out their work from home, thus multiplying their efforts and strains. The limitations imposed by the government during the first phase of lockdown (that is when data were collected) greatly hindered the women’s possibility to access to formal and informal support, thus potentially increasing feelings of isolation and fatigue which lead to the development of anxiety and mood disorders.

This study presents several limitations. First, it is a cross-sectional study that assesses expectant and postpartum women well-being only during the initial phase of the outbreak (the lockdown period). Thus, it is not possible to know whether the effects of the restrictive measures enforced by the Italian government will have long-lasting effects on the women’s well-being. Another methodological limitation concerns the data collection: data in this study have been collected online and this may affect the comparability with other studies using data collected in person. However, several other studies (Mott et al., 2011; Koletzko et al., 2015) have already used online surveys and did not report any difference in terms of the severity of the symptoms registered. Moreover, while the online modality might discourage some women from seeking help, it certainly helps in terms of reducing social desirability.

Another limitation has to do with the fact that the presence of a formal or informal support network has not been investigated in terms of its potentially moderating effect on the variables under investigation. Future studies might consider the possibility of investigating the effect of mediating and moderating variables. Finally, only a very limited number of women and their immediate family members resulted positive to Covid-19, thus making it impossible to conduct further analyses on the effects of contracting the virus on maternal well-being.

Despite these limitations, the present results clearly show that the pandemic emergency and the restrictions imposed on the population greatly impacted on prospective mothers’ and postpartum women’s well-being putting their mental health and emotional stability at stake. It is widely known that pregnancy and puerperium are extremely delicate moments in a woman’s life: not only women are called to re-negotiate their own identity and integrate the maternal role into their established role set (Rubin, 1975), they also enter a condition in which an increased attunement to their babies happens at the cost of their own self and often causes a general decline in their cognitive functions (Davies et al., 2018). Whether we call it “baby brain” (Davies et al., 2018) or “primary maternal preoccupation” (Winnicott, 1956), this particular state entails greater emotional instability and fragility as well as an increased need for protection. This is particularly true during stressful events, such as the ones experienced during a pandemic. In this perspective, it is key that health care facilities and medical staff keep under great consideration the role played by the support network in predicting both pregnant women and new-mothers’ well-being. Specifically, pregnant and postpartum women should be granted the presence of a companion of choice during delivery and in the first days after childbirth, as also suggested by the World Health Organization (2016). Not only granting such support results in better maternal outcomes but it has also been reported to favor bonding between all family members (Child and Family Research Partnership, 2014; Carvalho Coutinho et al., 2016). Moreover, the present study seems to suggest the need for developing specific interventions targeted at women who cannot benefit from the support of their partners or family.

## Data Availability Statement

The raw data supporting the conclusions of this article will be made available by the authors, without undue reservation.

## Ethics Statement

The studies involving human participants were reviewed and approved by Institutional Review Board of the Catholic University of Sacred Heart of Milan. The participants provided their written informed consent to participate in this study.

## Author Contributions

Both authors contributed to prepare the study design and reviewed, and approved the entire manuscript. SM wrote the introduction and the discussion sections. MA performed the analyses and wrote the methods and the results sections.

## Conflict of Interest

The authors declare that the research was conducted in the absence of any commercial or financial relationships that could be construed as a potential conflict of interest.
